# Vital Signs: Update on Zika Virus–Associated Birth Defects and Evaluation of All U.S. Infants with Congenital Zika Virus Exposure — U.S. Zika Pregnancy Registry, 2016

**DOI:** 10.15585/mmwr.mm6613e1

**Published:** 2017-04-07

**Authors:** Megan R. Reynolds, Abbey M. Jones, Emily E. Petersen, Ellen H. Lee, Marion E. Rice, Andrea Bingham, Sascha R. Ellington, Nicole Evert, Sarah Reagan-Steiner, Titilope Oduyebo, Catherine M. Brown, Stacey Martin, Nina Ahmad, Julu Bhatnagar, Jennifer Macdonald, Carolyn Gould, Anne D. Fine, Kara D. Polen, Heather Lake-Burger, Christina L. Hillard, Noemi Hall, Mahsa M. Yazdy, Karnesha Slaughter, Jamie N. Sommer, Alys Adamski, Meghan Raycraft, Shannon Fleck-Derderian, Jyoti Gupta, Kimberly Newsome, Madelyn Baez-Santiago, Sally Slavinski, Jennifer L. White, Cynthia A. Moore, Carrie K. Shapiro-Mendoza, Lyle Petersen, Coleen Boyle, Denise J. Jamieson, Dana Meaney-Delman, Margaret A. Honein, U.S. Zika Pregnancy Registry Collaboration, U.S. Zika Pregnancy Registry Collaboration, Jennifer Adair, Irene Ruberto, Dirk T. Haselow, Lucille Im, Wendy Jilek, Monica S. Lehmann, Richard Olney, Charsey Cole Porse, Karen C. Ramstrom, Similoluwa Sowunmi, Natalie S. Marzec, Karin Davis, Brenda Esponda-Morrison, M. Zachariah Fraser, Colleen Ann O'Connor, Wendy Chung, Folasuyi Richardson, Taylor Sexton, Meredith E. Stocks, Senait Woldai, Amanda M. Bundek, Jennifer Zambri, Cynthia Goldberg, Leah Eisenstein, Jennifer Jackson, Russell Kopit, Teresa Logue, Raphael Mendoza, Amanda Feldpausch, Teri Graham, Sylvia Mann, Sarah Y. Park, Kris Kelly Carter, Emily J. Potts, Taryn Stevens, Sean Simonson, Julius L. Tonzel, Shari Davis, Sara Robinson, Judie K. Hyun, Erin M. Jenkins, Monika Piccardi, Lawrence D. Reid, Julie E. Dunn, Cathleen A. Higgins, Angela E. Lin, Gerlinde S. Munshi, Kayleigh Sandhu, Sarah J. Scotland, Susan Soliva, Glenn Copeland, Kimberly A. Signs, Elizabeth Schiffman, Paul Byers, Sheryl Hand, Christine L. Mulgrew, Jeff Hamik, Samir Koirala, Lisa A. Ludwig, Carolyn Rose Fredette, Kristin Garafalo, Karen Worthington, Abubakar Ropri, Julius Nchangtachi Ade, Zahra S. Alaali, Debra Blog, Scott J. Brunt, Patrick Bryant, Amy E. Burns, Steven Bush, Kyle Carson, Amy B. Dean, Valerie Demarest, Elizabeth M. Dufort, Alan P. Dupuis II, Ann Sullivan-Frohm, Andrea Marias Furuya, Meghan Fuschino, Viola H. Glaze, Jacquelin Griffin, Christina Hidalgo, Karen E. Kulas, Daryl M. Lamson, Lou Ann Lance, William T. Lee, Ronald Limberger, Patricia S. Many, Mary J. Marchewka, Brenda Elizabeth Naizby, MaryJo Polfleit, Michael Popowich, Tabassum Rahman, Timothy Rem, Amy E. Robbins, Jemma V. Rowlands, Chantelle Seaver, Kimberley A. Seward, Lou Smith, Inderbir Sohi, Kirsten St. George, Maria I. Souto, Rachel Elizabeth Wester, Susan J. Wong, Li Zeng, Joel Ackelsberg, Byron Alex, Vennus Ballen, Jennifer Baumgartner, Danielle Bloch, Sandhya Clark, Erin Conners, Hannah Cooper, Alexander Davidson, Catherine Dentinger, Bisram Deocharan, Andrea DeVito, Jie Fu, Gili Hrusa, Maryam Iqbal, Martha Iwamoto, Lucretia Jones, Hannah Kubinson, Maura Lash, Marcelle Layton, Christopher T. Lee, Dakai Liu, Emily McGibbon, Morgan Moy, Stephanie Ngai, Hilary B. Parton, Eric Peterson, Jose Poy, Jennifer Rakeman, Alaina Stoute, Corinne Thompson, Don Weiss, Emily Westheimer, Ann Winters, Mohammad Younis, Ronna L. Chan, Laura Jean Cronquist, Lisa Caton, Leah Lind, Kumar Nalluswami, Dana Perella, Diane S. Brady, Michael Gosciminski, Patricia McAuley, Daniel Drociuk, Vinita Leedom, Brian Witrick, Jan Bollock, Marie Bottomley Hartel, Loraine Swanson Lucinski, Morgan McDonald, Angela M. Miller, Tori Armand Ponson, Laura Price, Amy E. Nance, Dallin Peterson, Sally Cook, Brennan Martin, Hanna Oltean, Jillian Neary, Melissa A. Baker, Kathy Cummons, Katie Bryan, Kathryn E. Arnold, Annelise C. Arth, Brigid C. Bollweg, Janet D. Cragan, April L. Dawson, Amy M. Denison, Eric J. Dziuban, Lindsey Estetter, Luciana Silva-Flannery, Rebecca J. Free, Romeo R. Galang, Joy Gary, Cynthia S. Goldsmith, Caitlin Green, Gillian L. Hale, Heather M. Hayes, Irogue Igbinosa, M. Kelly Keating, Sumaiya Khan, Shin Y. Kim, Margaret Lampe, Amanda Lewis, Cara Mai, Roosecelis Brasil Martines, Brooke Miers, Jazmyn Moore, Atis Muehlenbachs, John Nahabedian, Amanda Panella, Vaunita Parihar, Mitesh M. Patel, D. Brett Rabeneck, Sonja A. Rasmussen, Jana M. Ritter, Dominique C. Rollin, Jeanine H. Sanders, Wun-Ju Shieh, Regina M. Simeone, Elizabeth L. Simon, John R. Sims, Pamela J. Spivey, Helen Talley-McRae, Alphonse K. Tshiwala, Kelley VanMaldeghem, Laura Viens, Anne Wainscott-Sargent, Tonya Williams, Sherif Zaki

**Affiliations:** ^1^Division of Congenital and Developmental Disorders, National Center on Birth Defects and Developmental Disabilities, CDC; ^2^Division of Reproductive Health, National Center for Chronic Disease Prevention and Health Promotion, CDC; ^3^New York City Department of Health & Mental Hygiene; ^4^Oak Ridge Institute for Science and Education; ^5^Florida Department of Health; ^6^Texas Department of State Health Services; ^7^Division of High-Consequence Pathogens and Pathology, National Center for Emerging and Zoonotic Infectious Diseases, CDC; ^8^Massachusetts Department of Public Health; ^9^Division of Vector-Borne Diseases, National Center for Emerging and Zoonotic Infectious Diseases, CDC; ^10^New York State Department of Health; ^11^Virginia Department of Health; ^12^Epidemic Intelligence Service, CDC; ^13^Office of the Director, National Center for Emerging and Zoonotic Infectious Diseases, CDC; ^14^Office of the Director, National Center on Birth Defects and Developmental Disabilities, CDC.; Maricopa County Department of Public Health, Arizona; Arizona Department of Health Services; Arkansas Department of Health; Arkansas Department of Health; California Department of Public Health; California Department of Public Health; Center for Family Health; California Birth Defects Monitoring Program; California Department of Public Health; California Department of Public Health; California Department of Public Health; California Department of Public Health; Colorado Department of Public Health and Environment; Connecticut Department of Public Health; Connecticut Department of Public Health; Connecticut Department of Public Health; Connecticut Department of Public Health; Dallas County Health and Human Services; Dallas County Health and Human Services; Dallas County Health and Human Services; Dallas County Health and Human Services; Dallas County Health and Human Services; Delaware Division of Public Health; Delaware Division of Public Health; Office of Infectious Disease Epidemiology; Miami; Dade County Health Department; Florida Department of Health; Florida Department of Health; Orange County Health Department; Florida Department of Health; Palm Beach County Health Department; Florida Department of Health; Miami/Dade County Health Department; Florida Department of Health; Broward County Health Department; Florida Department of Health; Georgia Department of Public Health; Georgia Department of Public Health; Hawaii Department of Health; Hawaii Department of Health; Idaho Division of Public Health, CDC; U.S. Public Health Service; Indiana State Department of Health; Indiana State Department of Health; Louisiana Department of Health; Louisiana Department of Health; Maine Center for Disease Control and Prevention; Maine Department of Health and Human Services; Maryland Department of Health and Mental Hygiene; Maryland Department of Health and Mental Hygiene; Maryland Department of Health and Mental Hygiene; Maryland Department of Health and Mental Hygiene; Massachusetts Department of Public Health; Massachusetts Department of Public Health; Massachusetts General Hospital for Children; Massachusetts Department of Public Health; Massachusetts Department of Public Health; Massachusetts Department of Public Health; Massachusetts Department of Public Health; Michigan Department of Health and Human Services; Michigan Department of Health and Human Services; Minnesota Department of Health; Mississippi State Department of Health; Mississippi State Department of Health; State of Montana; Division of Public Health; Nebraska Department of Health and Human Services; Division of Public Health; Nebraska Department of Health and Human Services; Division of Public Health; Nebraska Department of Health and Human Services; New Hampshire Department of Health and Human Services; New Jersey Department of Health; New Jersey Department of Health; New Mexico State Department of Health; New York State Department of Health; New York State Department of Health; New York State Department of Health; Wadsworth Center; New York State Department of Health; Wadsworth Center; New York State Department of Health; New York State Department of Health; Wadsworth Center; New York State Department of Health; New York State Department of Health; Wadsworth Center; New York State Department of Health; Wadsworth Center; New York State Department of Health; New York State Department of Health; Wadsworth Center; New York State Department of Health; New York State Department of Health; Wadsworth Center; New York State Department of Health; Wadsworth Center; New York State Department of Health; Health Research Inc; New York State Department of Health; New York State Department of Health; Wadsworth Center; New York State Department of Health; Wadsworth Center; New York State Department of Health; New York State Department of Health; Wadsworth Center; New York State Department of Health; Wadsworth Center; New York State Department of Health; New York State Department of Health; Wadsworth Center; New York State Department of Health; New York State Department of Health; New York State Department of Health; Wadsworth Center; New York State Department of Health; New York State Department of Health; New York State Department of Health; New York State Department of Health; New York State Department of Health; New York State Department of Health; New York State Department of Health; New York State Department of Health; New York State Department of Health; Wadsworth Center; New York State Department of Health; Rockland County Department of Health; New York State Department of Health; Wadsworth Center; New York State Department of Health; Wadsworth Center; New York State Department of Health; New York City Department of Health & Mental Hygiene; New York City Department of Health & Mental Hygiene; New York City Department of Health & Mental Hygiene; New York City Department of Health & Mental Hygiene; New York City Department of Health & Mental Hygiene; New York City Department of Health & Mental Hygiene; New York City Department of Health & Mental Hygiene; New York City Department of Health & Mental Hygiene; New York City Department of Health & Mental Hygiene; New York City Department of Health & Mental Hygiene; New York City Department of Health & Mental Hygiene; New York City Department of Health & Mental Hygiene; New York City Department of Health & Mental Hygiene; New York City Department of Health & Mental Hygiene; New York City Department of Health & Mental Hygiene; New York City Department of Health & Mental Hygiene; New York City Department of Health & Mental Hygiene; New York City Department of Health & Mental Hygiene; New York City Department of Health & Mental Hygiene; New York City Department of Health & Mental Hygiene; New York City Department of Health & Mental Hygiene; New York City Department of Health & Mental Hygiene; New York City Department of Health & Mental Hygiene; New York City Department of Health & Mental Hygiene; New York City Department of Health & Mental Hygiene; New York City Department of Health & Mental Hygiene; New York City Department of Health & Mental Hygiene; New York City Department of Health & Mental Hygiene; New York City Department of Health & Mental Hygiene; New York City Department of Health & Mental Hygiene; New York City Department of Health & Mental Hygiene; New York City Department of Health & Mental Hygiene; New York City Department of Health & Mental Hygiene; New York City Department of Health & Mental Hygiene; New York City Department of Health & Mental Hygiene; North Carolina Department of Health and Human Services; Division of Public Health; North Dakota Department of Health; Division of Disease Control; Oklahoma State Department of Health; Pennsylvania Department of Health; Pennsylvania Department of Health; Philadelphia Department of Public Health; Rhode Island Department of Health; Rhode Island Department of Health; Rhode Island Department of Health; South Carolina Department of Health & Environmental Control; Division of Acute Disease Epidemiology; South Carolina Department of Health & Environmental Control; Division of Maternal and Child Health; South Carolina Department of Health & Environmental Control; Division of Acute Disease Epidemiology; South Dakota Department of Health DIS; Tennessee Department of Health; Tennessee Department of Health; Tennessee Department of Health; Tennessee Department of Health; Tennessee Department of Health; Tennessee Department of Health; Utah Birth Defect Network; Utah Department of Health; Utah Department of Health; Vermont Department of Health; Vermont Department of Health; Washington State Department of Health; Washington State Department of Health; West Virginia Office of Maternal, Child and Family Health; West Virginia Office of Maternal, Child and Family Health; Wyoming Department of Health; CDC; CDC; CDC; CDC; CDC; CDC; CDC; CDC; CDC; CDC; CDC; CDC; CDC; CDC; CDC; CDC; CDC; CDC; CDC,; ORISE; CDC; CDC; CDC; CDC; CDC; CDC; CDC; CDC; CDC; CDC; CDC; CDC; CDC; CDC; CDC; CDC; CDC; CDC; CDC; CDC; CDC; CDC; CDC; CDC; CDC; CDC; Carter Consulting; CDC; CDC

## Abstract

**Background:**

In collaboration with state, tribal, local, and territorial health departments, CDC established the U.S. Zika Pregnancy Registry (USZPR) in early 2016 to monitor pregnant women with laboratory evidence of possible recent Zika virus infection and their infants.

**Methods:**

This report includes an analysis of completed pregnancies (which include live births and pregnancy losses, regardless of gestational age) in the 50 U.S. states and the District of Columbia (DC) with laboratory evidence of possible recent Zika virus infection reported to the USZPR from January 15 to December 27, 2016. Birth defects potentially associated with Zika virus infection during pregnancy include brain abnormalities and/or microcephaly, eye abnormalities, other consequences of central nervous system dysfunction, and neural tube defects and other early brain malformations.

**Results:**

During the analysis period, 1,297 pregnant women in 44 states were reported to the USZPR. Zika virus–associated birth defects were reported for 51 (5%) of the 972 fetuses/infants from completed pregnancies with laboratory evidence of possible recent Zika virus infection (95% confidence interval [CI] = 4%–7%); the proportion was higher when restricted to pregnancies with laboratory-confirmed Zika virus infection (24/250 completed pregnancies [10%, 95% CI = 7%–14%]). Birth defects were reported in 15% (95% CI = 8%–26%) of fetuses/infants of completed pregnancies with confirmed Zika virus infection in the first trimester. Among 895 liveborn infants from pregnancies with possible recent Zika virus infection, postnatal neuroimaging was reported for 221 (25%), and Zika virus testing of at least one infant specimen was reported for 585 (65%).

**Conclusions and Implications for Public Health Practice:**

These findings highlight why pregnant women should avoid Zika virus exposure. Because the full clinical spectrum of congenital Zika virus infection is not yet known, all infants born to women with laboratory evidence of possible recent Zika virus infection during pregnancy should receive postnatal neuroimaging and Zika virus testing in addition to a comprehensive newborn physical exam and hearing screen. Identification and follow-up care of infants born to women with laboratory evidence of possible recent Zika virus infection during pregnancy and infants with possible congenital Zika virus infection can ensure that appropriate clinical services are available.

## Introduction

In response to the outbreak of Zika virus in the World Health Organization Region of the Americas and concerns about birth defects linked to Zika virus infection during pregnancy, CDC issued a travel notice on January 15, 2016, advising pregnant women to consider postponing travel to areas with active transmission of Zika virus. As part of the initial phase of the emergency response, CDC collaborated with state, tribal, local, and territorial health departments to establish the U.S. Zika Pregnancy Registry (USZPR) as an enhanced national surveillance system to monitor pregnancy and fetal/infant outcomes among pregnancies with laboratory evidence of possible recent Zika virus infection (*1*). The USZPR includes data on pregnant women and their infants at birth and at ages 2, 6, and 12 months.

The USZPR includes data from all 50 states, DC, and all U.S. territories except Puerto Rico; pregnancies in Puerto Rico are monitored separately by the Zika Active Pregnancy Surveillance System (*2*). To be included in the USZPR, either the pregnant woman, placenta, or fetus/infant must have laboratory evidence of possible recent Zika virus infection. Pregnant women in the United States and U.S. territories (with the exception of Puerto Rico) with laboratory evidence of possible recent Zika virus infection (regardless of whether they have symptoms) and the periconceptionally,* prenatally, or perinatally exposed infants born to these women are eligible to be included. The USZPR also includes infants with laboratory evidence of possible congenital Zika virus infection (regardless of whether they have symptoms or findings at birth) and their mothers.

This report updates the previous report (*3*) from the USZPR and provides data on pregnancies completed in the 50 U.S. states and DC from December 1, 2015 through December 27, 2016, reported to CDC from January 15, 2016, through March 14, 2017.^†^ Completed pregnancies include those of any length of gestation that end in a liveborn infant or a pregnancy loss. The baseline prevalence of defects consistent with those that have been observed with congenital Zika virus infection was approximately 2.9 per 1,000 live births in the pre-Zika years (*4*). The initial findings from the USZPR represent an approximate twentyfold increase in Zika virus–associated birth defects among pregnant women with laboratory evidence of possible recent Zika virus infection, with an approximate thirtyfold increase in brain abnormalities and/or microcephaly. Updated data in this report can also be compared with this benchmark (*3*,*4*).

## Methods

The USZPR defines laboratory evidence of possible recent Zika virus infection as 1) recent Zika virus infection detected by a Zika virus RNA nucleic acid test (NAT, e.g., reverse transcription–polymerase chain reaction [RT-PCR]) on any maternal, placental, or fetal/infant specimen or 2) detection of recent Zika virus infection or recent unspecified flavivirus infection by serologic tests on a maternal or infant specimen (i.e., either positive or equivocal Zika virus immunoglobulin M [IgM] AND Zika virus plaque reduction neutralization test [PRNT] titer ≥10, regardless of dengue virus PRNT value; or negative Zika virus IgM, AND positive or equivocal dengue virus IgM, AND Zika virus PRNT titer ≥10, regardless of dengue virus PRNT titer). Infants with positive or equivocal Zika virus IgM are included, provided a confirmatory PRNT has been performed on a maternal or infant specimen. The USZPR laboratory inclusion criteria are specified as “possible” recent Zika virus infection because the USZPR includes mother-infant pairs with serological evidence of a recent unspecified flavivirus infection, as well as those with laboratory-confirmed Zika virus infection.

Analyses were done on both the overall completed pregnancies in the USZPR from the 50 U.S. states and DC and a subset of completed pregnancies that demonstrated confirmed recent Zika virus infection (*5*,*6*). These are pregnancies in which the presence of Zika virus RNA in a maternal, placental, or fetal/infant specimen was documented by a positive NAT, or in which Zika virus IgM was positive or equivocal and Zika virus PRNT titer was ≥10 and dengue virus PRNT was <10.

Among symptomatic women, gestational timing of Zika virus infection was calculated using symptom onset date. Among asymptomatic women, the trimester of exposure was calculated using dates of travel to areas of active Zika virus transmission or sexual exposure. First trimester exposure was classified into two categories: 1) women with symptoms or exposure in the first trimester only^§^ (defined as first trimester or first trimester and periconceptional period); and 2) women with exposure during multiple trimesters including the first trimester. Estimates were not calculated for exposure in other trimesters because of small numbers. Pregnant women who did not have first trimester exposure might have had exposure in the periconceptional period only, second trimester, third trimester, or both the second and third trimester; for many women, the information on trimester of exposure was missing.

The Zika virus–associated birth defects (henceforth referred to as “birth defects”) were analyzed in two mutually exclusive categories: 1) brain abnormalities and/or microcephaly regardless of the presence of additional birth defects, and 2) neural tube defects and other early brain malformations, eye abnormalities, and other consequences of central nervous system dysfunction, among fetuses and infants without evident brain abnormalities or microcephaly (*7*). Clinical experts reviewed reported information to ensure that each fetus or infant with birth defects met the criteria of the USZPR case definition.

The proportion of fetuses or infants with birth defects among completed pregnancies was estimated among asymptomatic and symptomatic pregnant women, and women with first trimester exposure, using the Wilson score interval and 95% CI for a binomial proportion. Outcomes from multiple gestation pregnancies were counted once. Separate estimates were calculated for pregnancies with any laboratory evidence of recent Zika virus infection and for the subset of pregnancies with laboratory-confirmed recent Zika virus infection. For all liveborn infants with and without birth defects, the proportion who had any reported postnatal neuroimaging (cranial ultrasound, computed tomography, or magnetic resonance imaging) was calculated, as well as the proportion who had laboratory testing for Zika virus reported on an infant specimen. CDC released updated Interim Guidance for the Evaluation and Management of Infants with Possible Congenital Zika Virus Infection in August 2016 (*8*), which stated that postnatal neuroimaging and testing should be routine for all infants born to women with laboratory evidence of Zika virus infection during pregnancy; the proportion of infants with neuroimaging performed was calculated before and after this guidance was released.

## Results

**FIGURE 1:**
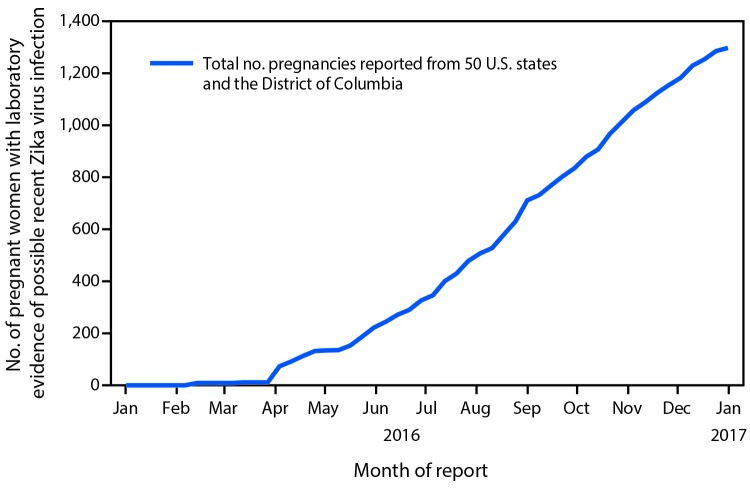
Cumulative number of pregnant women with laboratory evidence of possible recent Zika virus infection reported to the U.S. Zika Pregnancy Registry, by month of report — United States, January–December 2016 (n = 1,297)

From January 15 through December 27, 2016, a total of 1,297 pregnancies with possible recent Zika virus infection were reported to the USZPR from 44 states (Figure 1), including 972 completed pregnancies with reported outcomes (895 liveborn infants and 77 pregnancy losses). Among the completed pregnancies, 599 (62%) pregnant women were asymptomatic, 348 (36%) were symptomatic, and 25 (3%) had missing symptom information (Table 1).

**TABLE 1:** Pregnancy outcomes* for 972 women with completed pregnancies^†^ with laboratory evidence of possible recent Zika virus infection, by maternal symptom status and timing of symptom onset or exposure — U.S. Zika Pregnancy Registry, United States, December 2015–December 2016

Characteristic	Brain abnormalities and/or microcephaly (No.)	NTDs and early brain malformations, eye abnormalities, or consequences of CNS dysfunction without brain abnormalities or microcephaly (No.)	Total with ≥1 birth defect (No.)	Completed pregnancies (No.)	Proportion affected by Zika virus–associated birth defects, % (95% CI^§^)
**Any laboratory evidence of possible recent Zika virus infection** ^¶^					
**Total**	**43**	**8**	**51**	**972**	**5 (4–7)**
**Maternal symptom status**					
Symptoms of Zika virus infection reported	18	3	21	348	6 (4–9)
No symptoms of Zika virus infection reported	24	4	28	599	5 (3–7)
Unknown	1	1	2	25	—
Timing of symptoms or exposure**					
First trimester^††,§§^	13	1	14	157	9 (5–14)
Multiple trimesters including first	22	6	28	396	7 (5–10)
**Confirmed evidence of Zika virus infection** ^¶¶^					
**Total**	**18**	**6**	**24**	**250**	**10 (7–14)**
**Maternal symptom status**					
Symptoms of Zika virus infection reported	8	3	11	141	8 (4–13)
No symptoms of Zika virus infection reported	10	2	12	102	12 (7–19)
Unknown	1	0	1	7	—
Timing of symptoms or exposure**					
First trimester^††,§§^	8	1	9	60	15 (8–26)
Multiple trimesters including first	8	4	12	58	21 (12–33)

**Abbreviations:** CI = confidence interval; CNS = central nervous system; IgM= immunoglobulin M; NAT=nucleic acid test; NTD = neural tube defect; PRNT = plaque reduction neutralization test; RT-PCR = reverse transcription–polymerase chain reaction.

* Outcomes for multiple gestation pregnancies are counted once.

^†^ Includes live births, spontaneous abortions, terminations, and stillbirths.

^§^ 95% CI for a binomial proportion using Wilson score interval.

^¶^ Includes maternal, placental, or fetal/infant laboratory evidence of possible recent Zika virus infection based on presence of Zika virus RNA by a positive NAT (e.g., RT-PCR) or similar test, serological evidence of a recent Zika virus infection, or serological evidence of a recent unspecified flavivirus infection.

** Estimates were not calculated for exposure in other trimesters because of small numbers. Pregnant women who did not have first trimester exposure might have had exposure in the periconceptional period only (8 weeks before conception or 6 weeks before and 2 weeks after the first day of the last menstrual period), second trimester, third trimester, both the second and third trimester; many women were missing information on trimester of exposure.

^††^ First trimester is defined as last menstrual period +14 days to 13 weeks, 6 days (97 days).

^§§^ First trimester exposure includes women with exposure limited to the first trimester and women with exposure limited to the first trimester and periconceptional period.

^¶¶^ Includes maternal, placental, or fetal/infant laboratory evidence of confirmed Zika virus infection based on presence of Zika virus RNA by a positive NAT (e.g., RT-PCR) or similar test or serological results of IgM positive/equivocal with Zika PRNT ≥10 and dengue PRNT <10.

Birth defects were reported for 51 (5%) of the 972 completed pregnancies with laboratory evidence of possible recent Zika virus infection. The proportion was higher among completed pregnancies with confirmed Zika virus infection (24/250, 10%). Among completed pregnancies with confirmed Zika virus infection, 217 of 250 (87%) tested positive by RT-PCR, including 24 pregnancies with a fetus or infant with birth defects.

Birth defects were reported in similar proportions of fetuses/infants whose mothers did and did not report symptoms of Zika virus disease during pregnancy. Brain abnormalities and/or microcephaly were reported in 43 (84%) of 51 fetuses/infants with birth defects. Among pregnancies with confirmed Zika virus infection, brain abnormalities and/or microcephaly were reported in 18 (75%) of 24 fetuses/infants with birth defects. The 51 fetuses or infants with birth defects were from pregnancies with Zika virus exposure from the following 16 countries/territories with active Zika virus transmission: Barbados, Belize, Brazil, Cape Verde, Colombia, Dominican Republic, El Salvador, Guatemala, Guyana, Haiti, Honduras, Jamaica, Mexico, Puerto Rico, Republic of Marshall Islands, and Venezuela.

Birth defects were reported in a higher proportion of fetuses or infants whose mothers were infected during the first trimester of pregnancy. Among 157 pregnancies in which women had symptom onset or exposure to Zika virus infection during the first trimester, 14 (9%) fetuses/infants had reported birth defects (Table 1). When pregnancies with symptom onset or exposure during first trimester were limited to those with laboratory-confirmed Zika virus infection, nine (15%) of 60 completed pregnancies had reported birth defects.

**FIGURE 2:**
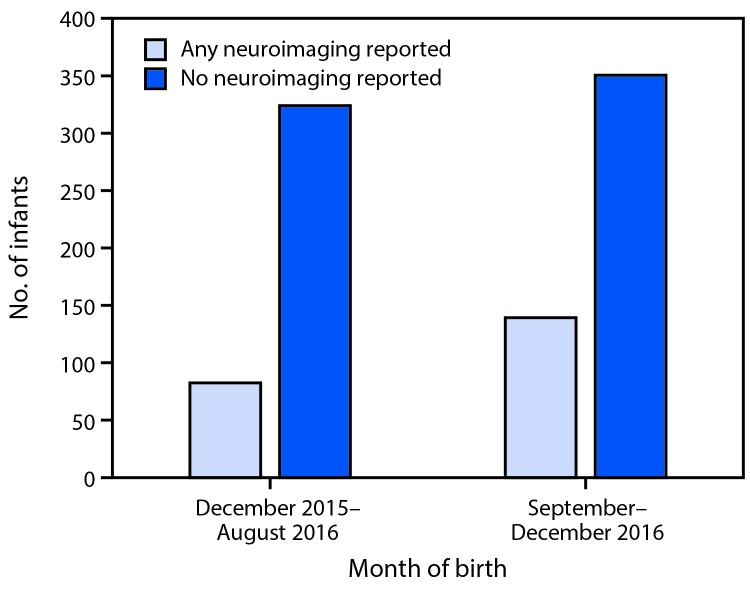
Postnatal neuroimaging for infants reported to the U.S. Zika Pregnancy Registry, by month of birth — United States, December 2015–December 2016

Among the 895 liveborn infants, postnatal neuroimaging results were reported to the USZPR for 221 (25%). Zika virus testing results of any specimen were reported for 585 (65%) infants; 94 (11%) of all 895 liveborn infants had positive Zika virus test results. Among the 45 liveborn infants with birth defects, 25 (56%) had positive infant Zika virus testing results reported, and 29 (64%) had postnatal neuroimaging reported to the USZPR (Table 2). Among the 850 liveborn infants without birth defects, 69 (8%) had positive infant Zika virus testing results reported, and 192 (23%) had postnatal neuroimaging reported to the USZPR. The percentage of infants reported to have received postnatal neuroimaging was 20% among 406 born through August 2016, and 28% among 489 born during September–December 2016, after the updated CDC guidance was released (*8*) (Figure 2).

**TABLE 2:** Postnatal neuroimaging* and infant Zika virus testing results for 895 liveborn infants in the U.S. Zika Pregnancy Registry — 50 U.S. states and the District of Columbia, 2016

Testing	No (%) liveborn infants		
	With birth defects	Without birth defects	Total
**Total**	**45 **	**850 **	**895**
**Neuroimaging**			
Any neuroimaging reported to USZPR	29 (64)	192 (23)	**221 (25)**
**Infant Zika virus testing**			
Positive test result on an infant specimen^†,§^	25 (56)	69 (8)	**94 (11)**
Negative infant test results among infants with ≥1 infant specimen reported as tested	17 (38)	474 (56)	**491 (55)**
No infant specimen test results reported to USZPR	3 (7)	307 (36)	**310 (35)**

**Abbreviations:** IgM= immunoglobulin M; NAT=nucleic acid test; RT-PCR = reverse transcription–polymerase chain reaction; USZPR = U.S. Zika Pregnancy Registry.

* Neuroimaging includes any cranial ultrasound, computed tomography, or magnetic resonance imaging test reported to the USZPR.

^†^ Positive infant tests included the presence of Zika virus RNA by a positive NAT (e.g., RT-PCR) and/or serological results of IgM positive/equivocal.

^§^ Infant specimens include serum, urine, blood, cerebrospinal fluid, cord serum, and cord blood.

## Conclusions and Comments

The number of pregnant women with laboratory evidence of possible recent Zika virus infection and the number of fetuses/infants with Zika virus–associated birth defects continues to increase in the United States. The proportion of fetuses and infants with birth defects among pregnancies with confirmed Zika virus infection at any time during pregnancy was more than 30 times higher than the baseline prevalence in the pre-Zika years, and a higher proportion of those with first trimester infections had birth defects (*4*). Although microcephaly was the first recognized birth defect reported in association with congenital Zika virus infection, Zika virus–associated brain abnormalities can occur without microcephaly, and neuroimaging is needed to detect these abnormalities (*9*). Neuroimaging is also used in other congenital infections to identify brain abnormalities; for example, neuroimaging findings in infants with congenital cytomegalovirus infection are correlated with neurodevelopmental outcomes (*10*). Postnatal neuroimaging is recommended for all infants born to women with laboratory evidence of Zika virus infection to identify infants with brain anomalies that warrant additional evaluation to ensure that appropriate intervention is provided (*8*). Based on data reported to the USZPR, the majority of these infants had not received recommended neuroimaging. In addition to infants with birth defects, complete follow-up and routine developmental assessment of all infants born to women with laboratory evidence of possible recent Zika virus infection is essential to help identify future outcomes potentially associated with congenital Zika virus infection and ensure that the referrals to appropriate support and follow-up care are made.

The findings in this report are subject to at least four limitations. First, selection bias might affect which pregnancies are reported to the USZPR, because pregnant women with symptoms of Zika virus disease might be more likely than asymptomatic women to be tested. Pregnant women with Zika virus exposure and prenatally detected fetal abnormalities or infants with birth defects might be more likely to be tested for Zika virus infection. In addition, pregnancies resulting in a loss might be more likely to have had a confirmed Zika virus infection and more likely to have the placenta or other pathologic specimens tested (*11*). However, it is also possible that birth defects in pregnancy losses, including stillbirths, have not been reported. Second, while CDC has worked closely with state and local health departments to obtain complete information, delays in reporting postnatal neuroimaging or infant Zika virus testing results are possible. In addition, some of the pregnancies included in the analysis were completed before CDC’s most recent infant guidance (*8*) was released, and thus, current recommendations for neuroimaging or testing might not have been implemented. Third, current testing methodologies are limited in that they can only identify recent Zika virus infections (*5*) and might miss those women who are tested when Zika virus RNA and/or IgM is no longer detectable; these pregnancies would not be included in the USZPR unless the fetus/infant or placenta has a positive Zika virus test result. Also, serologic testing cannot readily discriminate between flaviviruses because of crossreactivity (*5*); therefore, some pregnancies in the USZPR might have had a recent infection with a flavivirus other than Zika virus which could lead to an underestimate of the proportion of fetuses/infants affected. For this reason, in this report, analysis of the subset of pregnancies with laboratory-confirmed recent Zika virus infection was included. Finally, limited data are available about other maternal risk factors for birth defects, including genetic or other infectious causes, which might be causal factors for a few of the birth defects reported here.

These findings underscore the serious risk for birth defects posed by Zika virus infection during pregnancy and highlight why pregnant women should avoid Zika virus exposure and that all pregnant women should be screened for possible Zika virus exposure at every prenatal visit, with testing of pregnant women and infants in accordance with current guidance (https://www.cdc.gov/zika/pdfs/zikapreg_screeningtool.pdf) (*8*,*12*). Zika virus testing of infants is recommended for 1) all infants born to women with laboratory evidence of Zika virus infection in pregnancy and 2) infants with findings suggestive of congenital Zika syndrome born to women with an epidemiologic link suggesting possible transmission, regardless of maternal testing results. Infants without abnormalities born to women with an epidemiological link suggesting possible Zika virus exposure during pregnancy, and for whom maternal testing was not performed or was performed more than 12 weeks after exposure, should have a comprehensive exam. If there is concern about infant follow-up or maternal testing is not performed, infant Zika virus testing should be considered. The initial evaluation of infants should include a comprehensive physical examination, including a neurologic examination, postnatal neuroimaging, and standard newborn hearing screen. Additional evaluation might be considered based on clinical and laboratory findings, however routine developmental assessment is recommended as part of pediatric care (*8*). Based on initial USZPR reports, most infants born to women with laboratory evidence of possible recent Zika virus infection during pregnancy might not be receiving the recommended evaluation (e.g., postnatal neuroimaging). CDC is working with public health officials, professional societies, and health care providers to increase awareness of and adherence to CDC guidance for the evaluation and management of infants with possible congenital Zika virus infection. Identification and follow-up care of infants born to mothers with laboratory evidence of possible recent Zika virus infection during pregnancy and infants with possible congenital Zika virus infection can ensure that appropriate intervention services are available to affected infants.

**Key Points**

• In 2016, a total of 1,297 pregnancies with possible recent Zika virus infection were reported to the U.S. Zika Pregnancy Registry from 44 states.

• Approximately one in 10 pregnancies with laboratory-confirmed Zika virus infection resulted in a fetus or infant with Zika virus–associated birth defects.

• The proportion of fetuses and infants with Zika virus–associated birth defects was highest among those with first trimester Zika virus infections.

• Only 25% of infants from pregnancies with possible recent Zika virus infection reported receiving postnatal neuroimaging.

• Identification and follow-up care of infants born to mothers with laboratory evidence of possible recent Zika virus infection during pregnancy and infants with congenital Zika virus infection can ensure that appropriate intervention services are available to affected infants.

• Additional information is available at https://www.cdc.gov/vitalsigns/.

## Footnotes

* Periconceptional exposure is defined as maternal Zika virus infection during the 8 weeks before conception (6 weeks before and 2 weeks after the first day of the last menstrual period).

† Data on pregnancies reported to CDC by December 27, 2016; all data have been updated with additional information reported on these pregnancies through March 14, 2017. Completed pregnancies are limited to those with a pregnancy completion date on or before December 27, 2016.

§ First trimester is defined as last menstrual period +14 days to 13 weeks, 6 days (97 days).
